# Detection of body postures and movements in ambulatory adults with cerebral palsy: a novel and valid measure of physical behaviour

**DOI:** 10.1186/s12984-019-0594-9

**Published:** 2019-10-29

**Authors:** Everett A. Claridge, Rita J. G. van den Berg-Emons, Herwin L. D. Horemans, Wilma M. A. van der Slot, Nick van der Stam, Ada Tang, Brian W. Timmons, Jan Willem Gorter, Johannes B. J. Bussmann

**Affiliations:** 1 School of Rehabilitation Sciences, McMaster University, 1400 Main St. W, Hamilton, Ontario L8S 1C7 Canada; 2 CanChild Centre for Childhood Disability Research, McMaster University, 1400 Main St. W, Hamilton, Ontario L8S 1C7 Canada; 3 Department of Rehabilitation Medicine, Erasmus MC University Medical Centre, P.O. Box 2040, 3000 Rotterdam, CA Netherlands; 4 Rijndam Rehabilitation, Westersingel 300, 3015 Rotterdam, LJ Netherlands; 5 Child Health & Exercise Medicine Program, Department of Pediatrics, McMaster University, 1280 Main St. W, Hamilton, Ontario L8S 4L8 Canada

**Keywords:** Cerebral palsy, Activity monitor, Accelerometry, Physical activity, Physical behaviour, Objective measurement

## Abstract

**Background:**

Accurate measurement of physical behaviour is paramount to better understand lifestyle, health, and functioning, particularly in adults with physical disability as they may be at higher risk of sedentary lifestyle and subsequent negative health consequences. This study aimed: 1) to evaluate the criterion validity of a novel and clinically applicable activity monitor (AM, Activ8), in the detection of body postures and movements in adults with spastic cerebral palsy (CP); and 2) to evaluate the extent that the AM’s positioning affects validity.

**Methods:**

In this cross-sectional study, 14 ambulatory adults with CP [9 men; mean (SD) age, 35.4 (13.1) years] performed standardized activities while wearing three Activ8 monitors - frontolateral thigh (primary position), frontal thigh, and pant pocket - and being video recorded (criterion measure). AM activity output was compared to synchronized video recordings. Absolute (seconds) and relative [(video time–AM time)/mean time, %] time differences between methods were calculated. Relative time differences of < 10% were indicative of good validity. Comparison of AM attachment positions was completed using Spearman Rho correlation coefficients and Meng’s tests.

**Results:**

Criterion validity of the AM (frontolateral thigh) was good (average relative time differences: 0.25% for sitting, 4.69% for standing, 2.46% for walking, 1.96% for upright activity, 3.19% for cycling), except for running (34.6%). Spearman Rho correlation coefficients were greater between video/frontolateral thigh position than video/frontal thigh position and video/pant pocket position for body posture and movement categories sitting, standing, walking, and upright activity (*p* < 0.01 for all).

**Conclusions:**

The AM, positioned on the frontolateral thigh, demonstrated good criterion validity in ambulatory adults with CP. Though the Activ8 offers potential as an objective measure of physical activity, appropriate positioning is paramount for valid measurement.

## Introduction

Physical behaviour, which encompasses both physical activity (PA) and sedentary behaviour (SB), plays an important role in the health and functioning of adults [1–4]. Decreased PA and increased SB pose risks for negative health outcomes in adults, notably increased risk for cardiovascular disease and early mortality [5, 6]. To mitigate the possible health consequences of inactivity, clinicians are encouraged to promote PA to patients as a preventive health measure [7–9]. Promotion of physical behaviour is particularly important for adults with cerebral palsy (CP), as they have low levels of PA, increased SB, and reduced cardiorespiratory fitness [10–14]. Furthermore, as people with CP age, they undergo a decline in functioning [15, 16] that can further reduce PA and place them at risk of developing co-morbidities [17].

Knowledge of physical behaviour is essential for clinicians to effectively promote PA and limit SB in clinical populations [1, 9, 18]. Compared to self-report measures of physical behaviour, objective measures such as activity monitors (AMs) are generally considered more robust and sensitive to change [19]. Often used to assess physical behaviour over extended periods of time, AMs play an important role when evaluating interventions aiming to change physical behaviour [20, 21]. AMs, which include accelerometers, are objective in nature and can provide feasible measurement of PA in a free-living setting.

The recently developed Activ8 Physical Activity Monitor^a^ (Remedy Distribution Ltd., Valkenswaard, The Netherlands) offers a novel, objective method to measure PA. The main difference with other consumer monitors is that it provides information on distinct body postures and movements (P&Ms), whereas other AMs, such as the ActiGraph, mainly provide estimates of energy expenditure or evaluate time within PA intensities [22]. Additionally, the Activ8 is unobtrusive (34 × 30 × 10 mm, 20 g) and cost-friendly (€99), offering potential use in rehabilitation research and clinical settings. Activ8 users have the ability to view their recorded time spent in PA and SB on a personal computer or smart phone through the visually appealing dashboard (https://www.activ8all.com/app-dashboard/). Researchers and clinicians can add a coaching account, providing insight into their patients’ physical behaviour. Furthermore, features such as goal setting and an integrated feedback system may compel users to maintain a healthy and active lifestyle.

Preliminary evidence for criterion validity of the Activ8 AM exists in healthy adults (unpublished observations) and adults after stroke [23]. However, for individuals with atypical gait or movement patterns, like those with spastic CP, the validity of the device to detect body P&Ms needs to be determined before extending its use in larger research studies or in the clinical setting. Furthermore, though Activ8 was designed and calibrated for pocket placement, aforementioned preliminary evidence for validity of the Activ8 AM suggests that direct attachment to the thigh would be more advantageous due to issues regarding varying pocket positions, sizes and the potential for excessive movement. Therefore, the primary objective of this study was to evaluate the criterion validity of the Activ8 AM in the measurement of body P&Ms in ambulatory adults with spastic CP, the most common type of CP [24], compared to direct video observation (criterion measure). The secondary objective was to determine the extent that positioning of the Activ8 accelerometer on the thigh or in the pocket affected the validity of the device.

## Methods

### Participant Recruitment & Eligibility

A convenience clinical sample of fourteen adults with spastic CP [9 men; mean (SD) age, 35.4 (13.1) years] was recruited between February and June 2016. The sample size was based on comparable studies evaluating the validity of AMs with similar features, namely the VitaMove and the activPAL, in both children and adults with disability [25–27]. Participants were recruited through Rijndam Rehabilitation in Rotterdam, the Netherlands, by invitation from physicians once eligibility was determined and interest to participate was expressed. In addition, participants of past research studies who had indicated they would be interested in future research studies were contacted by mail and eligibility was confirmed by telephone. We aimed to recruit an equal distribution of participants across Gross Motor Function Classification System (GMFCS) levels I, II, and III.

The inclusion criteria were: ≥18 years of age; diagnosis of spastic CP; ambulatory, with or without the use of assistive devices; and physically able to perform the activities in the assessment protocol. Participants were excluded if they had: disabilities other than CP affecting daily PA; severe cognitive disorder; insufficient comprehension of either English or Dutch to follow instructions for testing, as determined during a screening telephone conversation; or orthopaedic surgery within the past 6 months. All participants provided written informed consent. The study was approved by the Medical Ethics Committee of Erasmus MC University Medical Centre, Rotterdam, NL.

### Measures

#### Activ8 activity monitor

**Fig. 1:**
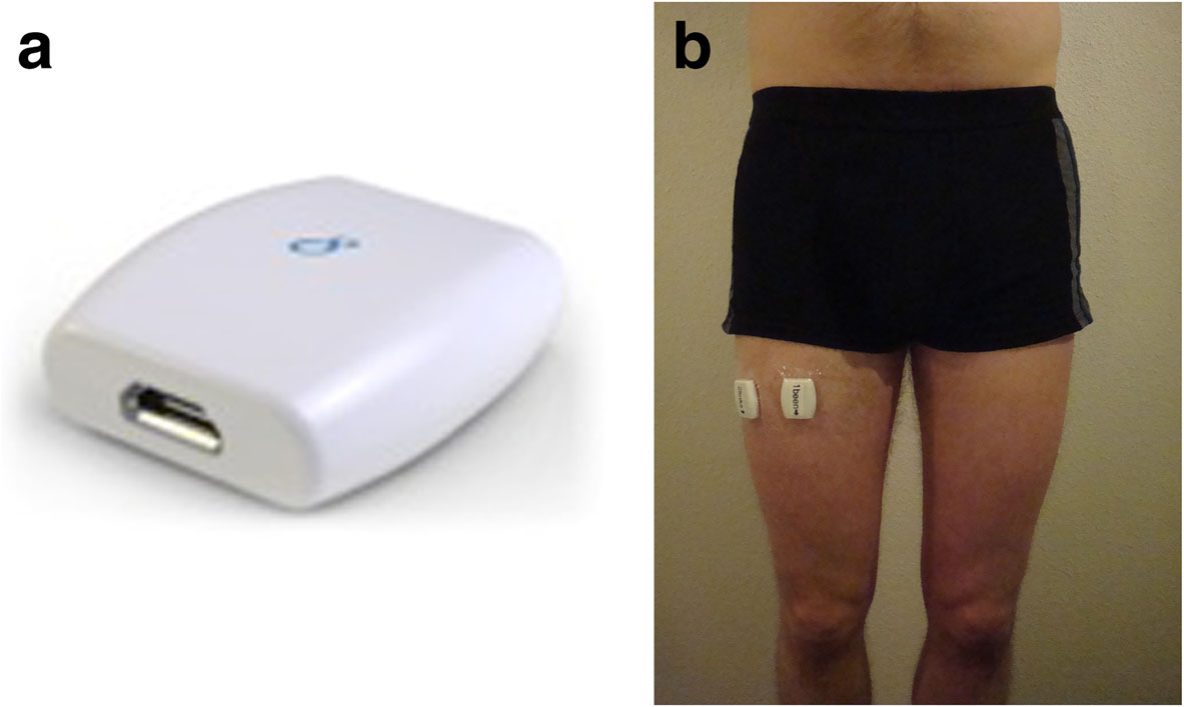
**a**. The Activ8 Physical Activity Monitor. **b**. Frontal (superior to patella) and frontolateral (2 cm laterally from frontal) thigh positioning of Activ8

The Activ8 AM (see Fig. 1a) contains a tri-axial accelerometer (MMA 8541, Freescale Semiconductor, Denver, USA) sensitive to high accelerations, all in the human activity spectrum (± 4.0 g in magnitude). The Activ8 has a sampling rate of 12.5 Hz and uses a 14-bit analog-to-digital converter. The Activ8 monitor then stores digitized acceleration samples using a First In, First Out (FIFO) data buffer that can hold 32 samples; therefore, each FIFO buffer holds 2.56 s of activity data.

Automated analysis of the angular position of the Activ8 AM, based on the signal from the z-axis accelerometer, as well as the vector magnitude acceleration (from x, y, and z-axes) allows raw acceleration samples to be converted into body P&M activity classes. These include the classes of non-wear/lying, sitting, standing, walking, bicycling, and running. The monitor was set to record body P&M data using a 5-s epoch, the lowest epoch setting available, allowing researchers to determine the amount of time spent in each Activ8 class. The Activ8 is unable to distinguish transient lying (< 5 min) from sitting, as the angular position of the monitor would remain the same for both static postures. Body P&M activity class data were downloaded using the professional Activ8 software (Version 2.1.0.22) and were used for comparison with video observation data.

#### Video observation

A handheld digital video camera was used as the reference method for detecting body P&Ms. Video recordings were watched two times, at half speed, by one researcher. These recordings were analysed and scored independently of the Activ8 data output. Using a 1-s time resolution, video recorded activity was assigned one of the following categories based on pre-defined definitions: non-wear; lying; sitting; standing; standing with movement; walking (including shuffling); stair climbing; running; bicycling; or transition (sit-to-stand/stand-to-sit).

The following steps were taken to minimize bias when scoring the criterion measure of video observation: 1) each 1-s video frame was coded based on rigid definitions of body P&M categories; 2) the video recording of the first participant was analysed by both author EC and an external researcher experienced with use of the Active8 – any discrepancies or areas of contention were discussed and a precedent was set for future issues encountered; and 3) any new issues encountered or periods of ambiguous body P&Ms during subsequent video recordings were highlighted and discussed between author EC and the external researcher until an agreed solution was reached.

### Monitor placement on the body

Participants were asked to wear three Activ8 monitors, two taped directly on the thigh with Tegaderm™ skin tape (frontolateral thigh and frontal thigh locations – see Fig. 1b) and one placed in the pocket of the trousers. The frontal thigh position was located 1/3rd the length of the thigh as measured from the greater trochanter to the patella. The frontolateral thigh location was located at the same height as the frontal thigh position but set 2 cm laterally from the thigh’s midline. This frontolateral position was our primary position as AM placement here more closely resembles the pant pocket position, for which the Activ8 was originally calibrated. All three monitors were placed at the side least affected by spasticity, or simply at the right side if there was no difference in spasticity among lower limbs.

### Procedure

All participants performed a series of activities according to a standardized protocol in a simulated free-living environment within the occupational therapy department and human movement laboratory at Erasmus MC, Rotterdam, NL. The Activity Protocol (see Table 1) consisted of meaningful activities representative of daily life in individuals with CP, as informed by the literature [25, 28, 29]. This protocol included both basic activities, involving just one posture or movement (e.g. standing, walking), and complex activities, involving a number of P&Ms (e.g. mopping the floor consists of standing and walking), and was completed in the same order each assessment. Participants were filmed for the duration of the assessment, while wearing the AMs. All activities were timed to be 80 s duration; less if the activity was completed before then (e.g. climbing two flights of stairs varied in duration). Start and stop times were noted during measurement, to ensure measures could be properly synchronized and compared. Body P&M activity class data were downloaded using professional Activ8 software upon completion of the assessment.

**Table 1:** Activity Protocol

Basic Activities	
Sitting	
Standing	
Walking on a flat surface	
-normal / comfortable pace	
-slower than normal	
-faster than normal	
Running	
Bicycling on a stationary bicycle	
- normal / comfortable pace	
- slower than normal	
- faster than normal	
Browsing through a magazine (sitting)	
Office work - Typing on a computer (sitting)	
Climbing the stairs (walking)	
Descending the stairs (walking)	
Complex Activities	
Donning and doffing a jacket	
Mopping the floor	
Unpacking and packing a grocery bag	
Folding laundry	
Washing dishes	
Ball sport exercise	
-dribbling and passing a soccer ball	
-dribbling and passing a basketball	
-throwing and catching a tennis ball	

Activ8 activity class data (5-s resolution) was then compared to the synchronized video observation (1-s resolution). Activ8 epochs do not display the order that numerous body P&Ms were performed. For example, a 5-s epoch may indicate that 2 s were spent standing and 3 s were spent walking, but it does not specify whether standing preceded walking or vice-versa. Therefore, each performed activity from the Activity Protocol was analysed as a whole. The first and last epoch sample were removed due to the potential overlap of the Activ8 epoch with the start and end time of each activity. Additionally, to allow comparison between measures, the following video classes were merged: lying to sitting and stair climbing to walking. The video class ‘standing with movement’ denoted ambiguous movement that fell between standing and walking classes (e.g. lunging to reach under a desk while mopping; see Table 2).

**Table 2:** Corresponding body P&M categories between video and Activ8 measures

Video	Activ8	
	*Basic activity protocol*	*Complex activity protocol*
Non-wear	Non-wear	Non-wear
Lying	Sitting	Sitting
Sitting		
Standing	Standing	Upright activity
Standing with movement	Standing OR Walking*	
Walking	Walking	
Stair climbing		
Running	Running	Running
Bicycling	Bicycling	Bicycling

*dependent on the activity itself

### Data analysis

Absolute time (|Activ8 time-video time|, seconds) and relative time [(|Activ8 time-video time|)/video time, %] differences were calculated for each P&M category for each Activ8 position, based on simple sum values of video and AM time. Additionally, median and quartile values of absolute and relative time differences were determined. For the primary analyses, an average relative difference of 10% or less between video and Activ8 measures was considered an acceptable level for criterion validity. This acceptable difference has been used in previous validation studies and is indicative of satisfactory validity [25, 30, 31].

Basic and complex activities (see Table 1) were analysed separately. “Standing with movement” time was allocated to the standing class during basic standing activities and to the walking class during basic walking activities. For complex activities, all standing, standing with movement, and walking time was allocated to a merged category labelled ‘upright activity’ (see Table 2).

To estimate agreement between measures on an individual level, Bland-Altman plots [32] were completed using data collected from the three Activ8 monitor positions. Plots compared the difference between measures (Activ8 time-video time, in sec) to the criterion, reference measure (video time) as recommended by Krouwer [33].

To compare data from the three Activ8 positions, the statistical program Stata (Version 13.1, Texas, USA) was used. Using the total time allocated to each class, Spearman Rho correlation coefficients were calculated between measures. The following categories were used to interpret correlation coefficients: 0.0 to 0.4 (poor to low agreement), 0.4 to 0.6 (low to moderate agreement), 0.6 to 08 (moderate to strong agreement), and 0.8 to 1.0 (very strong agreement) [34]. Meng’s tests [35] were then completed to determine whether correlation coefficients between video/frontolateral thigh Activ8, video/frontal thigh Activ8, and video/pant pocket Activ8 were statistically different.

## Results

Participant characteristics are displayed in Table 3. Half of the participating adults had unilateral spastic CP (hemiplegic distribution, *n* = 7) and half had bilateral spastic CP (diplegic or quadriplegic distribution, *n* = 7). As a result of lower-limb spasticity, four participants had a prominent crouched stance or a crouch gait pattern (i.e. > 20 degrees knee flexion). GMFCS levels were as follows: level I (*n* = 6), level II (*n* = 5), and level III (*n* = 3). Missing data resulted from lack of Activ8 equipment for participant number 3 and trousers lacking pockets for participant number 14.

**Table 3:** Participant Characteristics

Activ8 Monitors Worn							
Participant No.	Age (years)	Gender	Frontal Thigh	Frontolateral Thigh	Pant Pocket	Limb Distribution	GMFCS-E&R Level
1	23	F	X	X	X	Hemiplegia	I
2	26	M	X	X	X	Hemiplegia	I
3	28	F	X		X	Diplegia	III
4	23	M	X	X	X	Hemiplegia	I
5	48	M	X	X	X	Diplegia	II
6	46	M	X	X	X	Quadriplegia	III
7	55	M	X	X	X	Hemiplegia	I
8	58	M	X	X	X	Quadriplegia	II
9	21	F	X	X	X	Hemiplegia	I
10	43	M	X	X	X	Diplegia	II
11	24	F	X	X	X	Diplegia	II
12	29	M	X	X	X	Hemiplegia	II
13	27	M	X	X	X	Hemiplegia	I
14	45	F	X	X		Diplegia	III

The absolute and relative time differences between video and Activ8 measures for basic and complex activities are reported in Table 4. For the frontolateral thigh position, the average relative time differences between video and Activ8 methods were < 10% for detection of sitting (0.25%), standing (4.69%), walking (2.46%), and bicycling (3.19%), but > 10% for running (34.6%). Average relative time differences range from 2.92% (bicycling) to 34.9% (running) for the frontal thigh position and 0.41% (sitting) to 35.4% (running) for the pant pocket position.

**Table 4:** Total time, absolute time difference, and relative time difference between video observation and Activ8 AM

	Video time (sec)		Activ8 time (sec)		Absolute Time Difference (sec)		Relative Time Difference (%)	
Frontolateral Thigh	Median (Q1, Q3)	Simple sum (*n* = 13)	Median (Q1, Q3)	Simple sum (*n* = 13)	Median (Q1, Q3)	Total^b^ (*n* = 13)	Median (Q1, Q3)	Average^b^
* Basic Activities*								
Sitting	237 (232, 256)	3175	237 (232, 256)	3183	0 (0, 0)	8	0.00 (0, 0)	0.25
Standing		1066	78 (74, 81)	1016	3 (2, 7)	50	4.00 (2.4, 8.0)	4.69
Walking	82 (73, 86)	3706	286 (261, 311)	3615	4 (3, 8)	91	1.26 (1.0, 3.0)	2.46
Bicycling	291 (268, 302)	2598	229 (155, 232)	2515	0 (0, 7)	83	0.43 (0, 10)	3.19
Running	229 (221, 232) 69 (0, 72)	622	68 (3, 83)	837	5 (0, 16)	215	16.9 (8.8, 47)	34.6
* Complex Activities*								
Upright Activity^a^	460 (348, 544)	6072	463 (347, 544)	5953	1 (0, 9.5)	119	0.30 (0, 2.7)	1.96
Frontal Thigh	Median (Q1, Q3)	Simple sum (*n* = 14)	Median (Q1, Q3)	Simple sum (*n* = 14)	Median (Q1, Q3)	Total^b^ (*n* = 14)	Median (Q1, Q3)	Average^b^
* Basic Activities*								
Sitting	242 (231, 259)	3474	245 (233, 300)	3722	0 (0, 0.75)	248	0.00 (0, 0.29)	7.14
Standing		1139	75 (71, 78)	861	6 (1.5, 16)	278	7.30 (1.9, 21)	24.4
Walking	79 (75, 84)	3877	279 (250, 302)	3604	11 (1.8, 29)	273	3.68 (0.63, 14)	7.04
Bicycling	290 (278, 299)	2809	224 (208, 228)	2891	0 (0, 5.3)	82	0.00 (0, 2.7)	2.92
Running	226 (222, 232) 63 (0, 74)	628	64 (0, 94)	847	2.5 (0, 22)	219	19.2 (5.3, 42)	34.9
* Complex Activities*								
Upright Activity^a^	498 (332, 568)	6277	470 (281, 566)	5795	5 (0, 19)	482	1.30 (0.39, 3.34)	7.68
Pant Pocket	Median (Q1, Q3)	Simple sum (*n* = 13)	Median (Q1, Q3)	Simple sum (*n* = 13)	Median (Q1, Q3)	Total^b^ (*n* = 13)	Median (Q1, Q3)	Average^b^
* Basic Activities*								
Sitting	241 (232, 257)	3207	236 (223, 261)	3194	0 (0, 1)	13	0.00 (0, 0.6)	0.41
Standing		990	80 (75, 85)	1015	7 (1, 10)	25	8.33 (1.3, 57)	2.53
Walking	79 (74, 86)	3532	278 (244, 330)	3614	35 (7, 49)	82	11.2 (2.5, 34)	2.32
Bicycling	288 (252, 307)	2608	230 (107, 231)	2292	1 (0, 69)	316	5.75 (0, 44)	12.1
Running	230 (223, 231) 71 (0, 75)	624	65 (5, 95)	845	9 (0, 20)	221	26.7 (17, 66)	35.4
* Complex Activities*								
Upright Activity^a^	506 (242, 584)	6023	482 (223, 559)	5762	3 (0, 23)	261	2.01 (0.4, 6.4)	4.33

a. Upright Activity; the sum of Standing, Standing with Movement, and Walking categories

b. Based on simple sum values

**Fig. 2:**
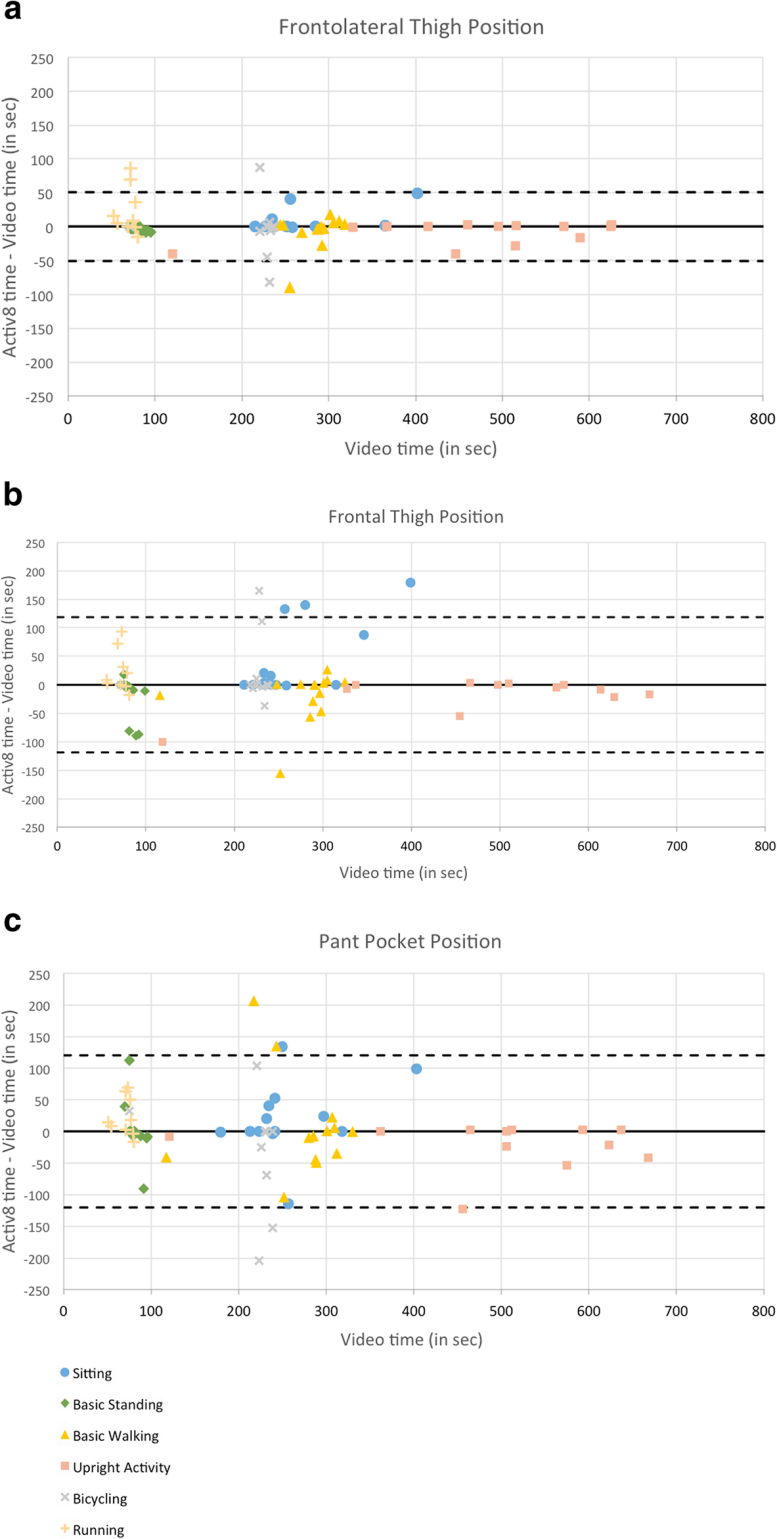
**a.** Frontolateral thigh position: Bland-Altman plot (Krouwer’s method) between video observation and Activ8 AM. **b**. Frontal thigh position: Bland-Altman plot (Krouwer’s method) between video observation and Activ8 AM. **c**. Pant pocket position: Bland-Altman plot (Krouwer’s method) between video observation and Activ8 AM. **a**, **b**, **c** legend: *See attached .png file*

Bland-Altman plots display biases between measurement tools and between-subject variability for each detected P&M (see Fig. 2). In these plots, the middle solid line represents the mean difference between methods and the wide dashed lines represent the upper and lower limits of agreement. The mean difference was smallest for the frontolateral thigh position (0.03, 95% CI = − 5.67 to 5.62 s) and larger for the frontal thigh (0.05, 95% CI = − 13.2 to 13.1 s) and pant pocket position (0.07, 95% CI = − 13.4 to 13.3).

Spearman Rho correlation coefficients between video observation and Activ8 AMs were calculated. For the frontolateral thigh position, correlation coefficients between Activ8 and video measures were moderate to strong ranging from 0.49 to 0.99. Correlation coefficients range from − 0.04 to 0.86 for the frontal thigh position and 0.14 to 0.79 for the pant pocket position. Meng’s tests (see Table 5) revealed that the correlation coefficients from video/frontolateral thigh Activ8 position were significantly larger than the correlation coefficients from video/front thigh Activ8 and video/pant pocket Activ8 for the P&M categories of sitting, basic standing, basic walking, and upright activity (*p* < 0.01 for all). The correlation coefficients from video/Activ8 measures were not different among AM wear locations for detection of bicycling or running.

**Table 5:** Meng’s Analysis: Comparison of Spearman Rho correlation coefficients between Activ8 positions and video observation

	Sitting	Basic Standing	Basic Walking	Upright Activity^a^	Bicycling	Running
r_1_	0.98	0.93	0.94	0.99	0.49	0.73
r_2_	0.86	−0.04	0.59	0.72	0.35	0.77
Z-score	2.96	3.60	2.88	4.82	0.42	1.36
*p*-value	.003*	< .001*	.004*	< .001*	.672	.174
r_1_	0.98	0.93	0.94	0.99	0.49	0.73
r_3_	0.48	0.14	0.37	0.79	0.39	0.74
Z-score	4.16	3.66	3.07	4.31	0.31	0.30
*p*-value	< .001*	< .001*	.002*	< .001*	.754	.763
r_2_	0.86	−0.04	0.59	0.72	0.35	0.77
r_3_	0.48	0.14	0.37	0.79	0.39	0.74
Z-score	2.44	0.46	0.87	1.00	0.14	1.03
*p*-value	.015*	.642	.384	.318	.886	.304

r_1_: correlation coefficient for video/frontolateral thigh Activ8; r_2_: correlation coefficient for video/frontal thigh Activ8; r_3_: correlation coefficient for video/pant pocket Activ8; a. Upright Activity: the sum of Standing, Standing with Movement, and Walking categories

* *p* < .05

## Discussion

This study evaluated the criterion validity of the Activ8 AM in the detection of body P&Ms during daily life activities in ambulatory adults with spastic CP. The low absolute and relative differences in time between video and Activ8 at the frontolateral thigh position suggest the Activ8 is an appropriate tool to detect body P&Ms in ambulatory adults with CP. One important caveat to this conclusion is the detection of running, which was poorly detected by the Activ8 (> 10% average relative time difference). The detection of body P&Ms from the frontal thigh and pant pocket positions was acceptable (< 10% difference) for the activity classes of sitting, walking, and upright activity but was not acceptable for standing (frontal thigh only), bicycling, and running.

An important characteristic of the Activ8 is the ability to distinguish between a set of body P&M categories. Most activity monitors assess the amount of PA or merely step counts, and not the type of PA [29–31]. However, systems such as the activPAL, Dynaport, and Vitaport/VitaMove also provide body P&M information. Compared to these systems the Activ8 is inexpensive, easy-to-use, and can provide direct feedback. A recent systematic review and meta-analysis has reported that AMs that provide users with ongoing feedback have moderately positive effects on levels of PA [36]. This suggests AMs like the Activ8 would be valuable components of PA promoting programs in the field of rehabilitation.

Since spasticity may lead to distorted body P&Ms that are different from other populations, we felt that a population-specific validation study in adults with spastic CP had to be performed. Comparison of our results with the results from other validity studies is difficult because of differences in protocol and data analysis; however, our results indicate comparable validity to other AMs that provide body P&M information in individuals with CP [25, 27, 37]. For example, like the activPAL [27, 37], the Activ8 demonstrated strong agreement for sitting, standing, and walking time compared to observation. However, the Activ8 distinguishes the additional body P&M categories of bicycling and running.

The Activ8, as a objective measure of physical behaviour in terms of body P&Ms, has been validated before in other populations including healthy adults (unpublished observations) and people after stroke [23]. As described in stroke [23], in adults with CP the Activ8 demonstrated appropriate detection of sitting, standing, walking, and cycling during basic activities and appropriate detection of upright activity during more complex daily activities. Additionally, this study further highlights the importance of AM placement on body P&M detection, which was a principal point of discussion in former Activ8 research as well [23].

Our results highlight important considerations with video observation and use of the Activ8 AM. Firstly, the distinction between standing and walking in the video was not always easy to make. In this study, movements that fell between standing and walking were given the classification ‘standing with movement’ and ranged from minor movements such as when standing restlessly to brief lunges such as when trying to reach a mop underneath a chair. ‘Standing with movement’ time was often detected as walking by the Activ8 and perhaps appropriately so. The majority of these ambiguous movements were fairly vigorous in nature and may require similar energy expenditure as walking. In this study, we chose to combine standing, walking, and ‘standing with movement’ activity categories into a pooled ‘upright activity’ category. Researchers and clinicians have suggested that it is important to promote breaks from SB, in addition to promoting PA, particularly for individuals with CP [8]. Any break from SB would be captured in the pooled ‘upright activity’ category during complex PA. Therefore, this pooled category may be appropriate when assessing free-living PA in adults with CP and would circumvent issues related to ‘standing with movement’ time.

In the current Activ8 classification algorithm, all movements surpassing a certain acceleration threshold will be categorized as running, regardless of the unit’s angular position, resulting in an overestimation of running time. This was seen in two participants. When asked to bicycle faster than normal, these two participants cycled at notably high intensities, leading to large vector magnitude accelerations. As a result, the correlation between Activ8 AM and video was low to moderate for the detection of bicycling (*r* = 0.49) and running (*r* = 0.73) despite the average relative time difference being well below 10% for bicycling (3.19%). Bicycling is an important activity, particularly in the Netherlands, and therefore inappropriate detection of vigorous bicycling as running time is a shortcoming of the Activ8. Based on the current classification algorithm, ‘running’ is not an appropriate title for this activity class and should be changed to ‘vigorous movement’ or ‘high intensity activity’ to better depict the detected movement.

Lastly, the positioning of the monitor is another important consideration, particularly for patients with hypertonicity, secondary musculoskeletal deformities, and deviated gait patterns. Muscle spasticity and contractures across joints may lead to a crouched stance/gait pattern [38, 39]. In this study, four participants with prominent crouched stances/gait patterns had standing frequently misclassified as sitting and walking misclassified as bicycling in the frontal thigh Activ8 location. Alternatively, positioning of the Activ8 AM on the frontolateral thigh resulted in more accurate detection of body P&Ms. At this frontolateral thigh location, gravitational/accelerative forces detected by the z-axis accelerometer are not as large as those detected by the frontal thigh monitor, leading to proper categorization of crouched stance and gait. One of the disadvantages with the frontolateral thigh position is that it is more difficult to standardize. If patients were asked to place the monitor on their own thigh, instructions to attach the monitor to the frontal thigh, along the midline of the thigh, are much simpler than instructions for the frontolateral thigh position.

### Study limitations

This study was only able to evaluate the validity of the Activ8 in a small sample of ambulatory adults with CP. However, when contrasting median and average relative time differences for the detection of each body P&M and inspecting the Bland-Altman plots, it is evident that gross misclassification of activity time by AM measures is only seen in one or two outliers. With a larger sample of adults, one could expect the average relative time difference to fall closer to the median, further supporting the validity of the Activ8 AM.

Another limitation to this study is use of only one rater to code video recordings. Previous validation studies using video (or direct) observation as criterion measures have used two raters to categorize observed activity for each 1-s frame, later comparing the scores of the raters and determining the inter-rater reliability [25, 27, 37]. In this study, potential bias when rating video recordings was minimized by using rigid definitions of body P&M categories as well as highlighting ambiguous video and discussing those video data with a researcher experienced in video coding.

The results of this study are limited to ambulatory adults with CP only, as validity will need to be established among other groups of ambulatory adults with impaired gait patterns as well as among non-ambulatory individuals. Future research to establish the validity of a Wheelchair Activ8 configuration would be invaluable to allow to researchers to assess the physical behaviour of a broad range of functional abilities, including non-ambulatory adults with neurological conditions such as CP, spinal cord injury, or stroke.

## Conclusion

The Activ8 shows promise as an objective measurement tool to assess physical behaviour in ambulatory adults with spastic CP. This device has shown adequate criterion validity in the detection of sitting, standing, walking, bicycling, and upright activity body P&M categories in a simulated free-living environment. Proper positioning of the AM is paramount to accurate body P&M detection, with attachment of the AM to the frontolateral thigh position resulting in better body P&M detection than frontal and pant pocket positions. The use of the Activ8 AM in larger clinical studies, inpatient, or outpatient settings should be explored further.

## Data Availability

The datasets used and/or analysed during the current study are available from the corresponding author on reasonable request.

## Abbreviations

AM: Activity monitor
CP: Cerebral palsy
FIFO: First in, first out
GMFCS: Gross motor function classification system
HPA: Habitual physical activity
P&Ms: Postures and movements
PA: Physical activity
SB: Sedentary behaviour
